# Loureirin B activates GLP‐1R and promotes insulin secretion in Ins‐1 cells

**DOI:** 10.1111/jcmm.16138

**Published:** 2020-12-10

**Authors:** Yanting Ding, Sijing Xia, Han Zhang, Qin Chen, Bing Niu

**Affiliations:** ^1^ School of Environmental and Chemical Engineering Shanghai University Shanghai China; ^2^ Shanghai Key Laboratory of Bio‐Energy Crops School of Life Sciences Shanghai University Shanghai China

**Keywords:** agonist, anti‐diabetic drugs, glucagon‐like peptide‐1 receptor, insulin, natural product

## Abstract

Loureirin B (LB) is a natural product derived from Sanguis draconis, which has hypoglycaemic effects. In order to research the possible target of LB in the treatment of diabetes, molecular docking was used to simulate the interaction between LB and potential targets, and among them, glucagon‐like peptide‐1 receptor (GLP‐1R) had the optimal results. Further, spectroscopy and surface plasmon resonance (SPR) experiments were applied to detect the interaction between LB and GLP‐1R. Ultimately, after GLP‐1R siRNA interfering the expression of GLP‐1R in Ins‐1 cell, the promoting insulin secretion of LB was weaken, which directly proved that GLP‐1R plays an important role. These results show that LB promotes insulin secretion of Ins‐1 cells through GLP‐1R. Hence, the strategy of LB as a prodrug will provide a potential approach for non‐peptide GLP‐1R agonist.

## 1 INTRODUCTION

Diabetes mellitus (DM), as a kind of metabolic disease, is characterized by the deficiency of insulin function and secretion, which leads to chronic hyperglycaemia, affecting the metabolism of protein, fat and carbohydrate.^1^, ^2^ DM patients mainly manifested as polyphagia, polydipsia, polyuria and weight loss. Type 2 diabetes mellitus (T2DM) accounts for 90‐95% of diabetic patients. Insulin resistance and β cell failure (decrease of β cell quality, glucose sensitivity and secretion ability) are the main characteristics.^3^, ^4^ DM is regarded by the World Health Organization (WHO) as one of the four main non‐communicable diseases (NCDs).^5^ Current treatment options for DM include the sole application of exogenous insulin or combining it with allopathic drugs. Those hypoglycaemic drugs decrease fasting blood glucose through many pathways, such as biguanides (metformin), sulphonylureas (glibenclamide) and alpha‐glucosidase inhibitors (acarbose and miglitol).^6^ Many efforts have been made to explore safe and effective anti‐T2DM drugs.^7^

Glucagon‐like peptide‐1 (GLP‐1) is a glucose‐dependent hormone that stimulates islet beta cells to secrete insulin in a glucose‐dependent manner.^8^ GLP‐1 receptor (GLP‐1R) belongs to the glucagon receptor B family and distributes in pancreatic cells, gastrointestinal tract, cardiovascular, lung and central nervous system.^9^ GLP‐1 continues to activate the GLP‐1R, which increases insulin secretion, beta‐cell proliferation and regeneration.^10^, ^11^ GLP‐1R is an important target for insulin secretion, the agonists of which are widely used in the treatment of DM.^12^ A series of GLP‐1R agonists, such as exdin‐4, liraglutide and lixisenatide, have been developed.^13^, ^14^ In addition to significantly reducing blood glucose, GLP‐1R agonists can also reduce weight, blood pressure, improving the function of β cell, which showed the potential to delay the progress of diabetes and reduce cardiovascular complications.^13^ GLP‐1R agonists can delay intestinal emptying, thereby increasing satiety, and suppressing appetite.^15^ Therefore, the weight‐loss effect of GLP‐1R agonists can improve both the quality of life of patients, and the metabolic abnormalities of obese T2DM.^16^ However, due to easily degrade by gastric juice and lose their activity in the body, these polypeptide drugs cannot be taken orally.^17^, ^18^ Therefore, the study of non‐peptide GLP‐1R agonists has become a hot topic.

*Resina draconis* is derived from the resin of *Dracaena cochinchinensis*, which has a variety of pharmacological effects, such as anti‐blood stasis, anti‐coagulation and hypoglycaemic.^19^, ^20^ Increasing researches are focused on how *Resina draconis* plays a role in the treatment of diabetes. Zhang et al reported that *Resina draconis* has good hypoglycaemic effect on rat hyperglycaemia model and increasing insulin secretion.^21^ The current research focuses on the hypoglycaemic mechanism of *Resina draconis*. In *Resina draconis*, the content of dihydrochalcones has relatively potential with hypoglycaemic effect such as loureirin B (LB).^19^ With the continuous determination of biomacromolecules and development of computer science, computer‐aided drug design has been widely used in the research and development of compounds. Through molecular docking to mimic the interaction of LB with various insulin secretion‐related targets, it was found that GLP‐1R may be a potential target for LB. Therefore, whether GLP‐1R is the target of LB was studied. The purpose of this study is to lay a foundation for the structural optimization of LB.

## 2 MATERIAL AND METHODS

### 2.1 Material

The recombinant protein GLP‐1R was obtained from Creative BioMart. Loureirin B (LB) was purchased from National Institutes for Food and Drug Control. The stock 0.01 mol/L LB solution was prepared with dimethyl sulphoxide (DMSO).

### 2.2 Molecular modelling and docking analyses

The extracellular domain of GLP‐1R was got from the PDB database (4ZGM, http://www.rcsb.org/pdb). The structure of LB was obtained from the PubChem database (https://pubchem.ncbi.nlm.nih.gov). The simulated interaction between LB and GLP‐1R was measured by Discovery Studio 4.1 (DS) (a suite of software for simulating small molecule and macromolecule systems).

The interactions between LB and GLP‐1R, DPP‐IV, ffar1 and GK were simulated by discovery studio 4.1 molecular docking software. The specific operation is as follows:

1. Open the receptor protein file downloaded from PDB database in DS software to prepare protein
  Tool∣Dock Ligands∣Prepare Protein.
2. Open the prepared protein file to obtain one or more potential docking sites
  Tool∣Define and Edit Binding Site.
3. Open the small molecule file downloaded from PubChem database in DS software to prepare ligand
  Tool∣Dock Ligands∣Prepare Ligands.
4. Running cdocker
  Tool∣Dock Ligands∣Docking Optimization∣Dock Ligands (CDOCKER).
  The main docking parameters are as follows:
  Top Hits:2000;Pose Cluster Radius:1;Random Conformations:10.
  Dynamics Steps:2000;Dynamics Target Temperature:310.15.
5. Analysis of docking results
  Tool∣View Interactions∣Display receptor‐ligand interactions.

### 2.3 2D and 3D fluorescence spectrometry

3D fluorescence spectroscopy: LB was added to the GLP‐1R solution with a concentration of 2.5 × 10^‐8^ mol/L (the concentration of the LB was 5 × 10^‐8^ mol/L). After mixed 5 minutes, the solution was measured by the three‐dimensional fluorescence spectra. Experimental parameters: excitation wavelength, 280 nm, emission wavelength, 290‐500 nm, slit width, 5 nm, and voltage, 950 V. The experiment was repeated 3 times.

2D fluorescence spectroscopy: LB was added to the GLP‐1R solution (2.5 × 10^‐8^ mol/L) 10 times continuously (the concentration of the LB was 0‐4.5 × 10^‐7^ mol/L). The equilibrium time of each reaction is 3min, and the emission spectrum of the mixture of GLP‐1R and LB at 290‐500 nm under the condition of excitation light of 280 nm is recorded. The experiment was repeated 3 times.

Fluorescence quenching constant Ksv and quenching rate constant Kq: The fluorescence spectra were obtained at different temperatures (300, 305, 310 K). When the excitation wavelength was 280 nm, the fluorescence intensity of the solution without LB was recorded as *F*_0_ when the reading emission light was 339 nm, and the fluorescence intensity of the solution with drugs was recorded as *F*. From Stern‐Volmer Equation ^22^:

(1) $$\frac{F_{0}}{F}=K_{sv}\left[C\right]+1=K_{q}\tau_{0}\left[C\right]+1$$

Using *F*_0_/*F* to plot the concentration of LB, the linear equation was obtained. The slope of the equation is the fluorescence quenching constant *K*_sv_;

(2) $$K_{SV}=K_{q}\tau_{0}$$

(τ_0_ represents the average lifetime of fluorescent molecules in the absence of loureirin, taking 10‐8s), *K*_q_ was obtained.^23^, ^24^

(3) $$lg\frac{\left(F_{0}‐F\right)}{F}=lgK_{a}+nlg\left[C\right]$$

The linear equation was obtained by drawing lg [(*F*_0_‐*F*)/*F*]. The slope of the equation was n and the intercept was lgKa.

(4) $${\mathrm{InK}}_{a}=‐\frac{H}{\mathrm{RT}}+\frac{S}{R}$$

(*T* was the absolute temperature, *R* was the gas constant 8.3145 J/mol *K*), the linear equation was obtained by drawing InKa for 1/*T*. The slope of the equation was ‐H/R, the intercept was S/R. The enthalpy, entropy and free energy were calculated.

(5) ΔG=ΔH‐TΔS

LB (0‐4.5 × 10^‐8^ mol/L) was gradually added to GLP‐1R (2.5 × 10^‐8^ mol/L). The synchronous fluorescence spectra of Δλ = 15 nm and Δλ = 60 nm were recorded.

Δλ = 15 nm: the emission wavelength was 250 nm, and the excitation wavelength was 235‐340 nm. Δλ = 60 nm: the emission wavelength was 295 nm, and the excitation wavelength was 235‐340 nm. Experimental parameters: slit width 5 nm, voltage 950 V.

### 2.4 Cell culture

Rat insulinoma Ins‐1 cells were purchased from American Type Culture Collection (ATCC). The cells were cultured in Dulbecco's modified Eagle's medium (DMEM) high‐glucose medium with 10% foetal bovine serum (FBS) (10 099 141, Gibco), 1% penicillin/streptomycin, 0.01 mol/L Hepes, 0.002 mol/L L‐glutamine, 0.001 mol/L sodium pyruvate, and 5 × 10^‐5^ mol/L β‐mercaptoethanol (M3148, Sigma) at 37°C in a humidified atmosphere with 5% CO_2_. The cells were seeded (5.0 × 10^5^ cells per mL) 2 days in culture dishes, and then fed with fresh medium. The experiment was repeated 3 times.

### 2.5 Assessment of insulin release and total insulin content

To measure insulin secretion, Ins‐1 cells were first seeded onto 24‐well plates and cultured for 48 h to approximately 80%‐90% confluence. Different concentrations of LB were mixed into cell culture medium. Cells treated with different concentrations of LB. The supernatants were collected from each well, and secreted insulin in which was determined using the Iodine [^125^I]− Insulin Radioimmunoassay Kit (North Biological Technology Research Institute of Beijing) according to the manufacturer's instructions.^25^ The experiment was repeated 3 times.

1. Put the reagents and samples required for the experiment at room temperature, dissolve the freeze‐dried products according to the operation requirements, and balance for at least 30 minutes before operation.
2. 60 × 12 mm polystyrene tube was numbered, and all experiments were repeated with double tubes.
3. 200 μL of temperature solution was added to each NSB tube, and 100 μL of temperature education solution was added to each standard ‘0’ tube.
4. Take 100 μL of standard products (a ~ f), quality control serum and sample of each concentration into the corresponding numbered test tube.
5. Each tube is added with 100 μL of marker working fluid.
6. Each tube was added with 100 μL antibody working liquid, and it was mixed well.
7. The temperature was raised at 37 ℃ for 2 hours.
8. Add 500 μL of separator to each tube and mix well.
9. After 15 minutes at room temperature, centrifuge 3500 rpm, and suck or pour off the supernatant after 20 minutes.
10. The recommended detection time: 1 minute.
11. The concentration of samples and quality control serum was read from the standard curve.

### 2.6 Cell viability

Cell viability was assessed with Cell Counting Kit‐8 (CCK‐8) (Sangon Biotech (Shanghai) Co., Ltd.). To determine cell viability, Ins‐1 cells were seeded at a density of 5000/well in 96‐well plates (100 μl/well). Then, the cells were cultured overnight and treated with culture medium containing 10^−5^‐10^‐9^ mol/L LB for different times. The absorbance values were measured at 450 nm using multifunctional microplate reader (PerkinElmer, Waltham). The experiment was repeated 3 times.

$$Cellviability\left(\%\right)=\frac{ODSample‐ODBlank}{ODSample‐ODBlank}\times 100\%$$

### 2.7 Real‐time PCR expression

The total RNA of Ins‐1 cells was extracted by TRIzol method (TRIzol‐based method for sample preparation) and measured by spectrophotometer. Complementary DNA was generated using the PrimeScript™ reverse transcript reagent Kit (Takara, Tokyo, Japan). The relative expression of genes associated with insulin secretion was determined by quantitative RT‐PCR (Bio‐Rad, Hercules, USA) according to the manufacturer's instruction. The experiment was repeated 3 times. The primer sequences used for PCR were as follows:

*Pdx1*,5′‐ AAATCCACCAAAGCTCACGC −3′ (forward) and 5′‐AAGTTGAGCATCACTGCCAGC‐3′(reverse). *Irs2*,5′‐CCCCAGTGTCCCCATCCT‐3′(forward) and 5′‐TTTCCTGAGAGAGACGTTTTCCA‐3′(reverse).

*GAPDH,*5′‐GGCAAGTTCAACGGCACAGT‐3’(forward) and 5′‐TGGTGAAGACGCCAGTAGACTC −3′(reverse).

1. After adding trizon to the sample, blow several times repeatedly to make the sample fully split. The protein nucleic acid complex was completely separated after being placed at room temperature for 5 minutes.
2. Add chloroform, add 0.2 ml chloroform every 1 ml trizon, cover the tube, shake violently for 15 seconds, and place at room temperature for 3 minutes.
3. After centrifugation at 4°C at 12 000 rpm for 15 minutes, the upper colourless aqueous phase (400 μL in 1 ml trizon) was removed and transferred into a new RNase free centrifuge tube.
4. Add equal volume of isopropanol into the extracted aqueous solution, mix it upside down and keep it at room temperature for 10 minutes.
5. The supernatant was centrifuged at 4°C 12 000 rpm for 10 minutes, and the supernatant was discarded.
6. 75% ethanol (prepared with water without RNase) was added to wash the precipitate. Wash the precipitate with 1 ml 75% ethanol every 1 ml trizon.
7. The supernatant was centrifuged at 4°C 12 000 rpm for 5 minutes, and the supernatant was discarded.
8. Centrifuging at 4°C 12 000 rpm for 30 seconds, carefully suck the remaining 75% ethanol and discard it.
9. Place at room temperature for 3 minutes and dry. 30‐100 μL of RNase free water was added to dissolve RNA, and the concentration of extracted RNA was determined. The obtained RNA can be stored in – 80°C refrigerator for subsequent experiments.

### 2.8 Western blot

Protein extracted from Ins‐1 cells after treatment was quantitated using Bradford's micro estimation assay. 20 μg samples were separated by sodium dodecyl sulphate‐polyacrylamide gel electrophoresis (SDS‐PAGE). Polyvinylidene fluoride (PVDF) membrane was utilized to electro‐transfer proteins, and then blocked with 5% non‐fat dry milk. And the membranes were incubated in different primary antibodies at 4°C overnight, including IRS‐2, pAKT(Ser473), FOXO1, H2A and GAPDH (CST). Then, the membrane was incubated with appropriate secondary antibody tagged with the horseradish peroxidase‐conjugated anti‐rabbit IgG secondary antibodies for 1 h at room temperature. Blots were detected and imaged by chemiluminescence using luminol as a substrate and ChemiDoc Imaging System (Bio‐Rad), respectively. The experiment was repeated 3 times.

### 2.9 SPR analysis

GE Biacore T200 instrument and GE S SA chips are used to complete the system. In the experiment, 0.05 mol/L NaOH and 1 mol/L NaCl pretreatment chips were injected three times and one minute at first, and the flow rate of coupling LB was 5 μg/L/min, which reduced the sample consumption. LB (1 × 10^‐5^ mol/L) was diluted with running buffer and then sampled and captured on the chip channel surface. In combination with the experiment, the reaction temperature was controlled at 25 (+0.1 degrees Celsius) and the flow rate was set at 30 μL/min. The detection channel (LB) subtracts the reference channel in real time to eliminate the volume effect caused by non‐specific binding and buffer. Phosphate buffer saline (PBS) (0.05 mol/L, pH 7.4) acts as running buffer. Different concentrations of LB were prepared and their binding with GLP‐1R was detected by SPR experiments.^26^ The experiment was repeated 3 times.

### 2.10 siRNA transfection

Ins‐1 cells were transferred to 6‐well plates and cultured at 37°C in a humidified atmosphere with 5% CO_2_. SiRNA was transfected with Lipofectamine™ 2000 Transfection Reagent according to the manufacturer's instructions.

### 2.11 Statistical analysis

Statistical significance was determined using the one‐way analysis of variance (ANOVA). All analyses were performed using SPSS Statistics ver. 19.0 (SPSS Inc). *P* values of less than .05 indicated statistical significance. Results are presented as mean ± SD.

## 3 RESULTS

### 3.1 Effects of LB on insulin secretion in Ins‐1 cells

**Figure 1:**
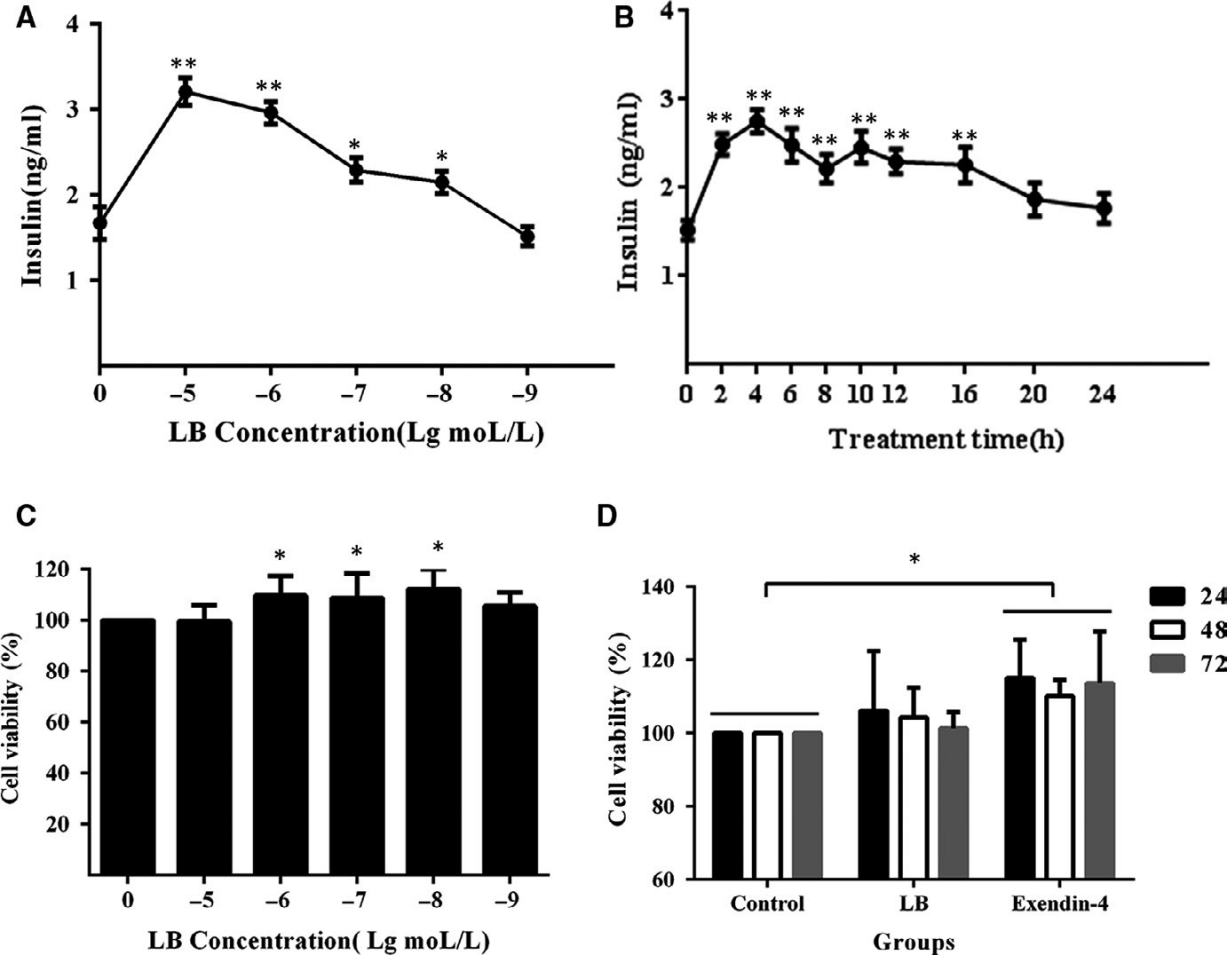
(A) Effects of different concentration of LB on insulin secretion. Ins‐1 cells were treated with LB at different concentrations (10^‐5^, 10^‐6^, 10^‐7^, 10^‐8^, 10^‐9^ mol/L) for 4 h. (B) Effects of different treatment time of LB on insulin secretion. Ins‐1 cells were treated with LB (10^‐6^ mol/L) for different time (0‐24 h). The levels of insulin were measured with immunoradiometric assay method. (C) Effects of different concentration of LB on the viability of Ins‐1 cells. Ins‐1 cells were treated with LB at different concentrations (10^‐5^, 10^‐6^, 10^‐7^, 10^‐8^, 10^‐9^ mol/L) for 24 h. (D) Effects of different treatment time of LB on the viability of Ins‐1 cells. Ins‐1 cells were treated with LB (10^‐6^ mol/L) for different time (24‐72 h). The cell activity was measured by the method of CCK‐8. (n = 3, *P < .05, **P < .01, ***P < .005)

Ins‐1 cells were treated with LB at different concentrations and time, and insulin secretion and cell viability were measured (Figure 1). The results showed that insulin secretion of Ins‐1 increased with the increment of LB (Figure 1A, C). When the concentration of LB was higher than 1 × 10^‐8^ mol/L, insulin secretion of Ins‐1 was significantly higher than control group. However, there is no significant increase of insulin secretion by continuing reduce LB. It is worth noting that LB significantly promotes insulin secretion in the range of 10^‐5^‐10^‐9^ mol/L, while no cytotoxicity in Ins‐1 cells.

Ins‐1 cells were treated with LB for different time. The results (Figure 1B, D) showed measurements of the insulin secretion and cell viability. The tendency of insulin secretion was first rising and then gradual; and reached the optimal value after 4 hours of treatment, about 2.78 ± 0.24 ng/ mL.

### 3.2 Interactions between LB and GLP‐1R

#### 3.2.1 Simulation of the interaction between LB and GLP‐1R by molecular docking

**Figure 2:**
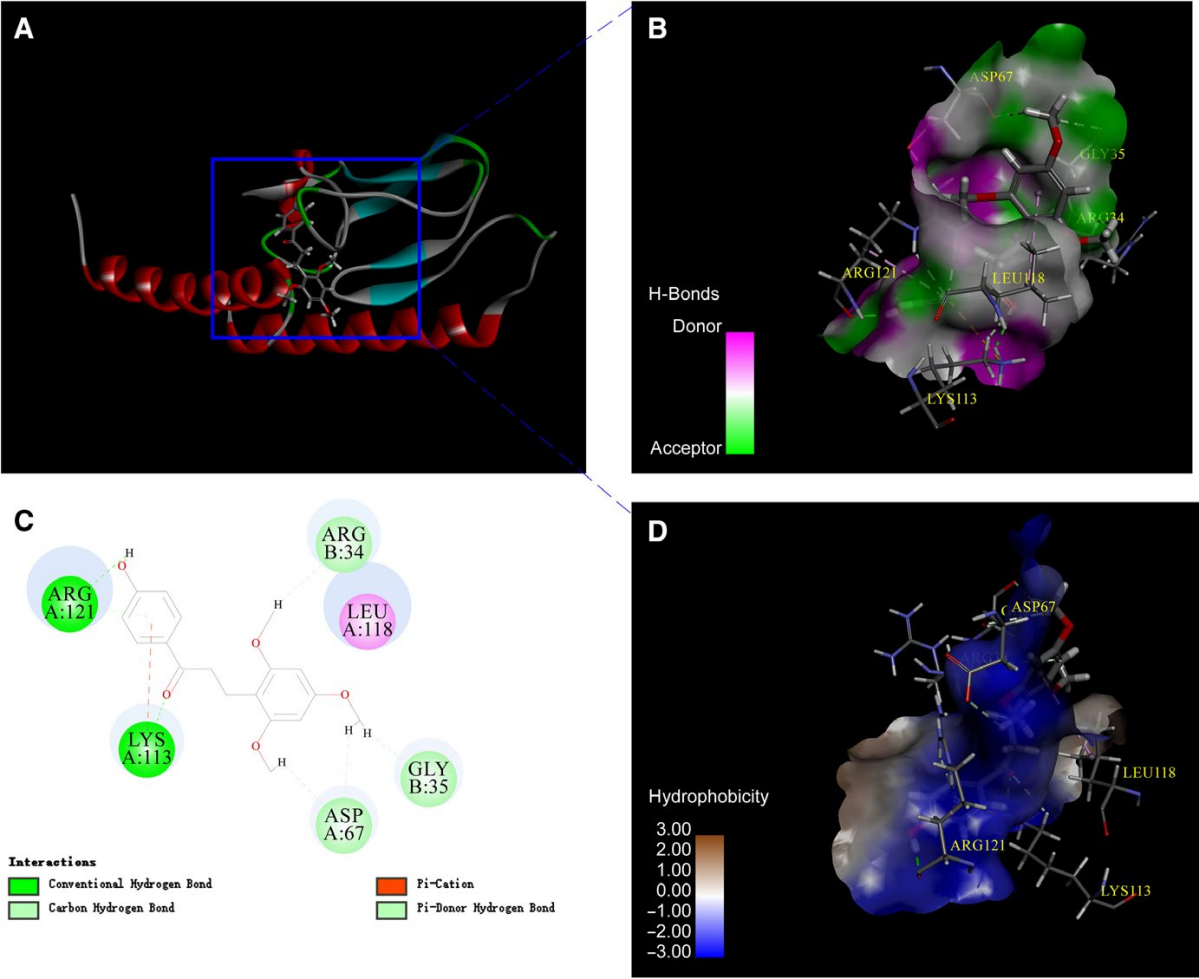
Molecular docking diagram of LB interaction with GLP‐1R.(A) 3D structure of interaction between LB and GLP‐1R.(B) Hydrogen bonding between LB and GLP‐1R.(C) 2D structure of interaction between LB and GLP‐1R.(D) Hydrophobic interaction between LB and GLP‐1R

After confirming the insulin secretion‐promoting function of LB, relative targets associated with the effect were evaluated. GLP‐1R, FFAR1, GK and DPP‐IV are general targets for anti‐diabetic drugs. Discovery studio 4.1 was used to simulate the binding ability between LB and targets. CDOCKER as the docking method showed the highest accuracy, which indicated the best ability to reproduce the natural binding mode. The attribute of ‘‐CDOCKER_ENERGY’ was used as a metric to measure the binding affinity. From Table 1 and Fig. S1, the ‐cDocker Energy values of the interaction are 40.4163, 34.5128, 31.0388 and 25.4087, respectively. The results explained that the combination of LB and GLP‐1R are stability than others. It was speculated that LB may interact with GLP‐1R. Further analysis of the interaction type showed that hydrogen bonding is the mainly type between LB and GLP‐1R (Figure 2).

**Table 1:** Molecular docking results of LB and DPP‐IV, FFAR1, GK, GLP‐1R

Targets	‐cDocker energy	Bond length	Acting force	From	To
DPP‐IV	25.4087	2.85493	Hydrogen Bond	B:LYS523:HE1	LB:O16
		2.37264	Hydrogen Bond	LB:H34	B:GLN586:O
		2.49159	Hydrogen Bond	LB:H39	B:GLY424:O
		5.30472	Hydrophobic	LB	B:PRO426
GK	31.0388	4.78337	Electrostatic	A:ARG205:NH1	LB:O17
		2.69057	Hydrogen Bond	A:ARG205:HE	LB:O17
		2.58088	Hydrogen Bond	A:ARG205:HH12	LB:O22
		1.74203	Hydrogen Bond	A:ARG205:HH21	LB:O17
		2.19633	Hydrogen Bond	A:ARG205:HH22	LB:O22
		2.9287	Hydrogen Bond	A:ASN305:HD22	LB:O18
		2.31156	Hydrogen Bond	A:HIS352:HE2	LB:O16
		2.10915	Hydrogen Bond	A:GLY448:HN	LB:O17
		2.45706	Hydrogen Bond	A:VAL447:HA	LB:O17
		2.66581	Hydrogen Bond	LB:H42	A:ALA94:O
		5.39622	Hydrophobic	LB	A:ALA94
GLP‐1R	40.4163	1.7705	Hydrogen Bond	A:THR29:HT3	LB:O16
		3.09601	Hydrogen Bond	LB:H43	A:TRP87:O
		2.33015	Hydrogen Bond	LB:H43	A:LEU89:O
		2.80355	Hydrogen Bond	A:THR29:HB	LB:O16
		2.6848	Hydrogen Bond	LB:H39	B:GLU16:OE2
		2.63173	Hydrogen Bond	LB:H41	A:GLU128:OE2
		2.86651	Electrostatic	A:LYS38:HZ2	LB
FFAR1	34.5128	1.75076	Hydrogen Bond	A:LYS1035:HZ1	LB:O16
		2.66067	Hydrogen Bond	A:GLY1110:HN	LB:O22
		2.16996	Hydrogen Bond	A:ARG1137:HH12	LB:O17
		1.90192	Hydrogen Bond	A:ARG1137:HH22	LB:O17
		4.89805	Electrostatic	A:GLU1022:OE2	LB

#### 3.2.2 Determination of the interaction between LB and GLP‐1R by SPR

**Figure 3:**
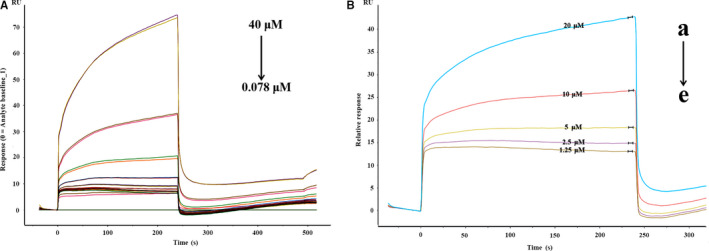
SPR sensorgrams corresponding to different concentrations LB and GLP‐1R. (A) LB was diluted with running buffer to different concentrations and then sampled and captured on the chip channel surface (4 × 10^‐5^ mol/L‐7.8 × 10^‐8^ mol/L). (B) The detection channel LB (a‐e:2 × 10^‐5^ mol/L‐1.25 × 10^‐6^ mol/L) was sampled and captured on the chip channel surface

Meanwhile, a series of SPR experiments were performed to evaluate the combination of different concentrations of LB with GLP‐1R. The interaction between 10 concentrations of LB and GLP‐1R (Figure 3A) was examined; five of them were selected for further analysis. GLP‐1R was coupled to the chip and flowed with different concentrations of LB. As shown in Figure 3B, the RU value increased in a gradient with the increase of LB. When the LB concentration was 20um, the RU value reached 44, while the control was 0, which excluded the influence of the buffer on the experimental results. The binding constant calculated by SPR is 5.56 × 103 L/mol, which further proved that LB can interact with GLP‐1R.

#### 3.2.3 Types of Interaction between LB and GLP‐1R

**Figure 4:**
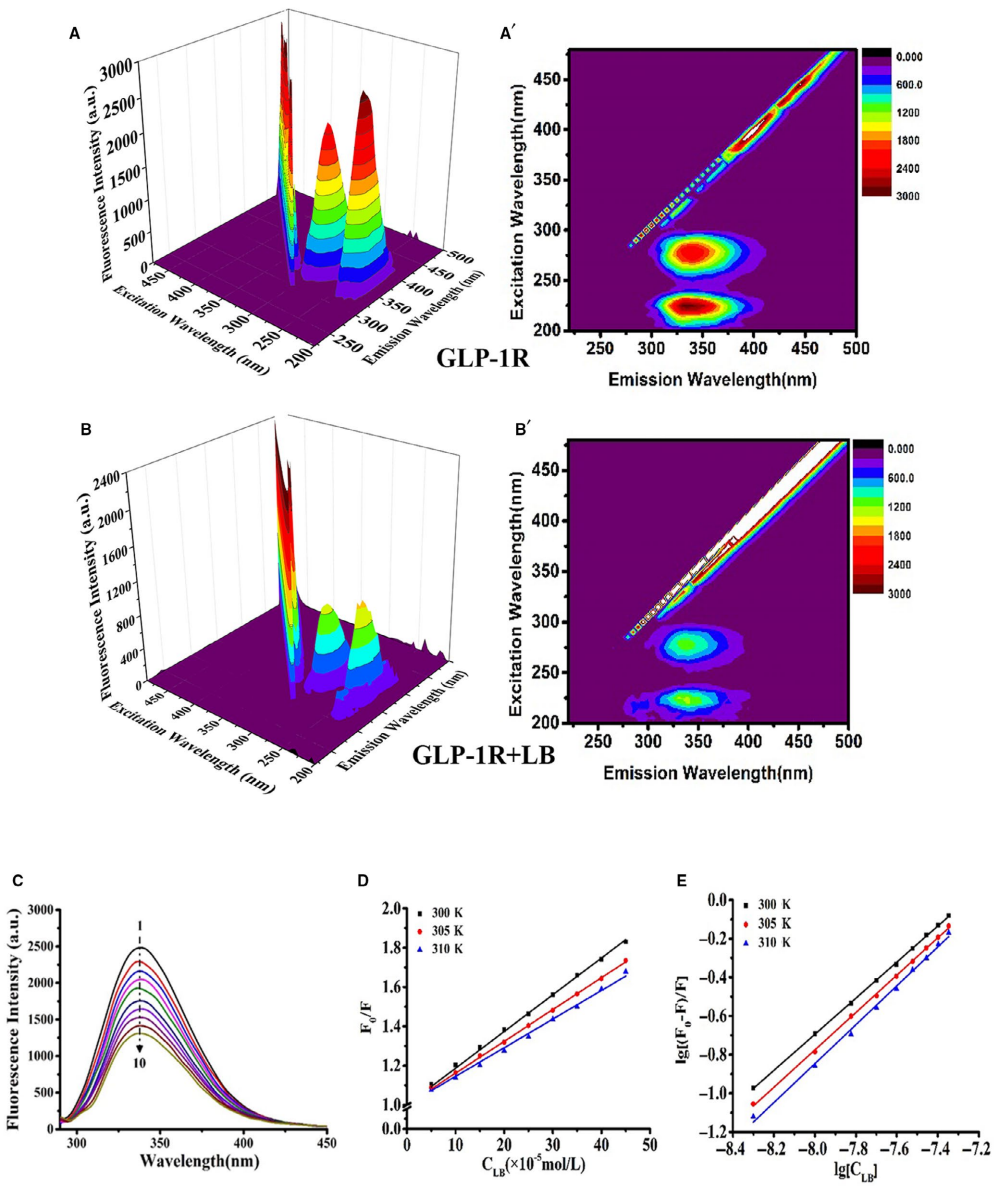
Three‐dimensional fluorescence spectra, two‐dimensional fluorescence spectra, fluorescence quenching Stern‐Volmer diagram and quenching double reciprocal curve of the interaction between LB and GLP‐1R. (A‐B) 3D Fluorescence Spectra of LB and GLP‐1R. (C) 2D fluorescence spectroscopy, GLP‐1R' concentration constant (2.5 × 10^‐8^ mol/L) while varying the LB concentration from 0 mol/L to 4.5 × 10^‐7^ mol/L (line 1‐10, increasing 5 × 10^‐8^ mol/ L in turn). (D) Stern–Volmer curves of GLP‐1R fluorescence quenched LB. (E) Double‐log plots of LB quenching effect on GLP‐1R's fluorescence

Based on the mentioned results, the interaction between LB and GLP‐1R was further explored by three‐dimensional(3D) fluorescence spectroscopy. The 3D fluorescence spectrum of GLP‐1R and LB reactions is shown in Figure 4A‐B. There were two fluorescence peaks of GLP‐1R at 347 nm when the excitation wavelength is 230 nm and 280 nm. The fluorescence peak position remained unchanged after adding LB, while the fluorescence intensity decreased. As is well known, the fluorescence of 230 nm excitation light and 345 nm emission light is related to the structure of protein polypeptide chain and the aromatic amino acid residues (tyrosine, tryptophan, phenylalanine). Thus, the interaction between LB and GLP‐1R was confirmed. We speculated that LB might interact with tyrosine, tryptophan and phenylalanine residues in GLP‐1R and subsequently affect the peptide chain structure of GLP‐1R.

Two‐dimensional (2D) fluorescence spectra of LB reacted with GLP‐1R were recorded and are shown in Figure 4C. With the addition of LB, the fluorescence intensity of the solution gradually decreased, indicating that LB interacted with GLP‐1R.

In order to investigate the type of fluorescence quenching of GLP‐1R by LB, the Stern‐Volmer diagram of fluorescence quenching was plotted according to Equation 1 (Figure 4D). Then according to Equation 2, the fluorescence quenching constant Ksv and quenching rate constant Kq are calculated (Table 2). As can be seen from Table 2, Kq was more than 2 × 10^10^ mol/L. The result demonstrated that the fluorescence quenching of LB to GLP‐1R was due to the static quenching caused by the complex reaction with the GLP‐1R protein in the ground state. The interaction type between LB and GLP‐1R was further analysed based on the results of fluorescence spectroscopy. ΔH and ΔS of the interaction of LB and GLP‐1R at different temperatures were calculated according to Equation 4, and then ΔG is obtained from Equation 5 (Table 2). According to the thermodynamic laws of Ross et al, LB and GLP‐1R mainly have hydrogen bonds in the process of binding, which was consistent with the molecular docking results. ΔG of the interaction between LB and GLP‐1R was negative, demonstrating that the reaction proceeded spontaneously. Based on Equation 3, the double reciprocal curve of the fluorescence quenching of GLP‐1R caused by LB (Figure 4E) was plotted. The binding constant Ka between LB and GLP‐1R was calculated (Table 2).

**Table 2:** Thermodynamic parameters, fluorescence quenching constant Ksv and quenching rate constant Kq of LB on GLP‐1R

T(K)	ΔH(KJ/mol)	ΔG(KJ/mol)	ΔS(J mol^‐1^ K^‐1^)	K_sv_(10^6^ L/mol)	K_q_(10^14^L/mol)	Ka (10^3^ L/mol)
300		−41.33		1.87	1.87	6.95
305	−76.69	−40.74	−117.82	1.62	1.62	6.77
310		−40.15		1.46	1.46	7.05

#### 3.2.4 Effect of LB on the conformation of GLP‐1R

**Figure 5:**
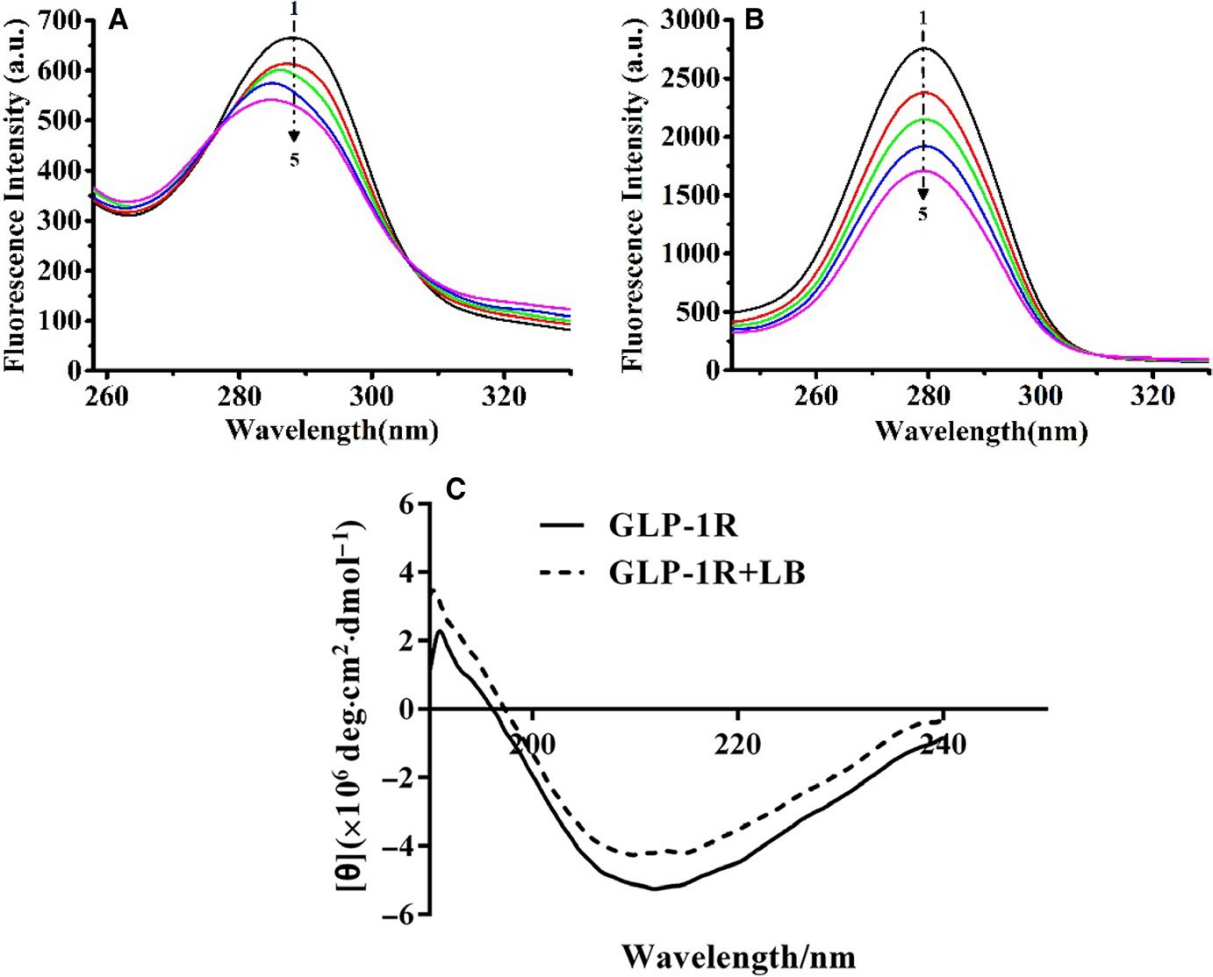
Synchronous fluorescence of the interaction between LB and GLP‐1R. (A) Δλ = 15 nm; (B) Δλ = 60 nm; (C) Circular dichroism of the interaction between LB and GLP‐1. (Concentration of GLP‐1R is 2.5 × 10^‐8^ mol/L; Lines 1‐5 denotes the concentration of LB in the range of 0‐4 × 10^‐8^mol/ L, respectively (increasing 1 × 10^‐8^ mol/ L in turn))

The above experimental results showed that LB may affect tyrosine, tryptophan and phenylalanine in GLP‐1R. Here, the effect of LB on protein conformation was determined based on synchronized fluorescence. When the spacing between excitation wavelength and emission wavelength (Δλ) is 15 and 60 nm, respectively, the synchronous fluorescence spectra represent the spectral characteristics of Tyr and Trp. When Δλ = 15 nm, fluorescence quench and blue shift was observed while adding LB, which indicated that LB has an effect on tyrosine residues in GLP‐1R (Figure 5A). When Δλ = 60 nm, the addition of LB also quenches the fluorescence of GLP‐1R, but the maximum excitation wavelength does not shift (Figure 5B). Comparing the two sets of synchronous fluorescence spectra, it is suggested that the fluorescence quenching of GLP‐1R under the action of LB is mainly caused by the interaction between LB and Trp residues in GLP‐1R. When Δλ = 15 nm, the blue‐shifted of the maximum excitation wavelength of the solution is due to the change of the microenvironment of the Tyr residue in the GLP‐1R peptide chain under the action of LB. This change affects the hydrophobicity of GLP‐1R protein surface. Hence, it is considered that LB changes the conformation of GLP‐1R by binding to it.

Circular Dichroism (CD) was an important method for studying the secondary structure of proteins and calculating the content of different secondary structures in proteins. Three‐dimensional fluorescence and ultraviolet results showed the changed secondary structure of GLP‐1R causing by LB. Then, circular dichroism spectra were used to analyse the secondary structure of GLP‐1R. The CD spectra of GLP‐1R changed obviously after adding LB, which indicated that LB had an effect on the secondary structure of GLP‐1R (Figure 5C). As Table 3 shown, LB could increase the contents of alpha‐helix and beta‐fold in GLP‐1R; and decrease the contents of beta‐angle and irregular curl.

**Table 3:** The secondary structure of GLP‐1R by SELCON (%)

Structure	Regular α‐helix	Irregular α‐helix	Regular β‐folding	Irregular β‐folding	β‐turn	Random coil
GLP‐1R	20.2 (1)	12.8 (1)	14.2 (1)	10.5 (1)	16.6 (1)	25.7 (1)
GLP‐1R + LB	35.3 (1.75)	17.1 (1.34)	7.3 (0.51)	4.6 (0.44)	14.3 (0.86)	21.3 (0.83)

### 3.3 The role of GLP‐1R in LB promoting insulin secretion

**Figure 6:**
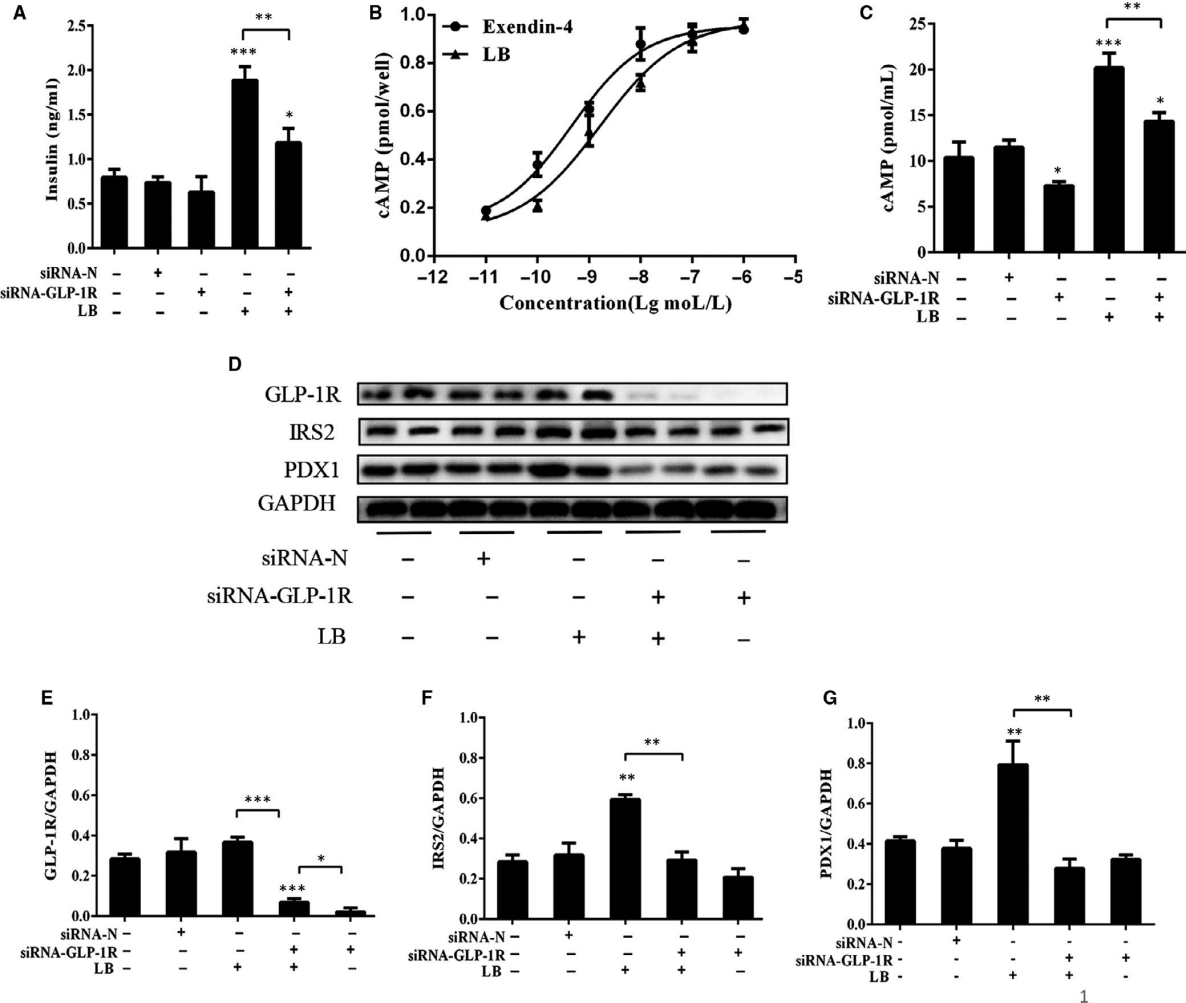
(A) The role of GLP‐1R in promoting insulin secretion of LB. Ins‐1 cells were interfered with siRNA‐GLP‐1R for 48 hours and treated with LB (10^‐6^ mol/L) for 2 h. The levels of insulin were measured with immunoradiometric assay method. (B) Effect of different concentration of LB and exendin‐4 on cAMP level. Ins‐1 cells were treated with concentration of LB (10^‐11^‐10^‐5^ mol/L) for 30 min. Intracellular cAMP levels were determined using a cAMP assessment kit according to the manufacturer's instructions. (C) The effect of GLP‐1R on cAMP level. At 48 hours post‐transfection, ins‐1 cells were treated with LB(10^‐6^ mol/L) for 30 min. Intracellular cAMP levels were determined using a cAMP assessment kit according to the manufacturer's instructions. (D‐G) At 48 hours post‐transfection, Ins‐1 cells were treated with LB(10^‐6^ mol/L) for 2 h. IRS2 and PDX1 protein were analysed by Western blotting. (n = 4, *P < .05, **P < .01, ***P < .005)

Since the Ins‐1 cell insulin secretion promoted by the complexation of LB and GLP‐1R obtained from molecular docking and fluorescence results, we used siRNA‐GLP‐1R to interfere Ins‐1 cells for 48 hours. The insulin secretion of Ins‐1 cells was determined, as shown in Figure 6A. After treating the cells with LB, the insulin secretion of Ins‐1 cells was significantly increased. However, interfered GLP‐1R, the insulin secretion of Ins‐1 cells turned to decrease. It was further proved that LB promoted insulin secretion by activating GLP‐1R. The content of cAMP was also determined and shown in Figure 6B‐C. The half‐maximum effective concentration (EC_50_) of the LB was 1.619 × 10^‐9^ mol/L. The results illuminated that LB increased the cAMP level in Ins‐1 cells. Interestingly, after interfered GLP‐1R, the addition of LB still increased the cAMP level, which indicated that LB could also increase the cAMP level in Ins‐1 cells through other pathways. Despite, it still could confirm that LB activated GLP‐1R to up‐regulated cAMP. After LB treatment, the expression of RS2 and PDX1 increased significantly (Figure 6D‐F), while the interference of GLP‐1R leads to the significant decrease of IRS2 and PDX1 protein expression. Therefore, it could be concluded that LB up‐regulated PDX1 protein expression by activating GLP‐1R.

## 4 DISCUSSION

The *Resina draconis* is a famous herb medicine used for a variety of applications including blood stasis, oxidative stress, inflammation, tumours and immune suppression. It also reported that the molecular and cellular basis of the immunomodulatory activities are mediated by *Resina draconis* and its active component LB. Direct inhibition of Kv1.3 in T cells by LB might be the cellular and molecular basis of dragon's blood‐mediated immunosuppression. In the last decade, researchers have reported the idea that similar to type 1 diabetes, type 2 diabetes is also an autoimmune disease and have found evidence that insulin resistance may be the result of immune system cells attacking the body's tissues.^27^ Studies have shown that *Resina draconis* has good hypoglycaemic effect and can enhance insulin secretion.^21^ The main component, LB was investigated whether it promotes insulin secretion. The results showed that LB significantly promotes the secretion of insulin in Ins‐1 cells. The results exhibited that the insulin secretion increased continuously at 2‐4 h and turn to decrease gradually after 4‐12 h. This phenomenon means that LB has a long half‐life. When reached the peak, insulin secretion will not increase indefinitely but decrease slowly, which improves the safety of use and reduces the side effects like hypoglycaemia.

GLP‐1R, as a kind of G protein‐coupled receptor, is an important target for the treatment of DM, which mediates the action of GLP‐1. At present, GLP‐1R agonists have not identified any adverse side effects. Molecular docking was used to simulate the interaction between LB and GLP‐1R extracted from *Resina draconis*. The main purpose of this paper is to find the potential target of LB. Molecular simulation results showed that GLP‐1R is the potential target of LB. SPR detection confirmed the interaction between LB and GLP‐1R. Fluorescence spectra and circular dichroism prove that LB has an effect on the conformation of GLP‐1R. In cell experiments,, GLP‐1R was demonstrated as the target of LB. The docking results showed through hydrogen bond may be the main interaction between LB and GLP‐1R; and the results of LB‐GLP‐1R docking were better than other target simulations. The docking conclusions provided clues for the potential interaction of determination of LB targets. Fluorescence spectroscopy is a general method for explaining the interaction between drugs and biomolecules. Three‐dimensional fluorescence spectroscopy and synchronous fluorescence spectroscopy indicated that LB may change the conformation of Tyr and Trp through hydrogen bonding and van der Waals force, thus changing the structure of the peptide chain, resulting in a change in the conformation of GLP‐1R. This is consistent with the molecular docking simulation. Moreover, the overlap of the fluorescence emission spectrum of GLP‐1R and the ultraviolet absorption spectrum of LB shows that a possibility of energy transfer between LB and GLP‐1R, which also provides evidence for the interaction between LB and GLP‐1R.

Although confirmed the interaction between LB and GLP‐1R, it was not enough to prove GLP‐1R is target of LB. Interestingly, when GLP‐1R was interfered, the secretion of insulin in the LB treatment group was decreased, but still significantly up‐regulated compared with the control. Subsequently, the intracellular cAMP level was measured, and the results showed that LB significantly increased the intracellular cAMP level of Ins‐1. After the interference of GLP‐1R, the cAMP of the LB treatment group also decreased, but still higher than the control group. These results showed that in addition to GLP‐1R, LB may promote insulin secretion and increase cAMP levels through other receptors or pathways. In other words, GLP‐1R is not the only target of LB to promote insulin secretion. Looking for other targets of LB will be our goal in future research. At present, there are many insulin‐stimulating genes related to G protein‐coupled receptors, such as FOXO1, PDX1, IRS2 and GCK. Based on the pathway proposed by Kitamura T, et al PDX1 and IRS2 are considered as the important genes in insulin secretion. The outcomes showed that LB up‐regulates the expression of IRS2 and PDX1 genes and proteins. The expression of IRS2 and PDX1 protein was down‐regulated significantly after interfered GLP‐1R. It can be speculated that LB promotes insulin secretion by activating GLP‐1R and up‐regulating PDX1 by signal transduction. Since good insulin secretion‐promoting effect of LB, new compounds derived from LB might be potential drug against T2DM. PDX1 is not only related to insulin secretion, but also to the proliferation of islet cells. Cell activity experiments have confirmed that LB can promote the activity of Ins1 cells. Therefore, we believe that LB may also have an effect on the proliferation of islet cells. This is the main direction of our follow‐up work. Optimizing molecular structure, improving water solubility, and investigating the mechanism of action of drugs will be the focus of further research in the future.

Although the interaction between LB and GLP‐1R has been supported using fluorescence spectrometry and CD, these experiments could not provide the information of interactional specificity between LB and the potential prodrugs. Therefore, the docking models presented in this study belong to predictive modelling, which can be applied to any type of unknown event, regardless of when it occurred.^28^, ^29^, ^30^ However, in order to obtain a more accurate docking model, interactional specificity between molecules needs to be obtained. NMR spectroscopy is a powerful tool to study specificity of biomolecule‐biomolecule ^31^, ^32^, ^33^, ^34^ or biomolecule‐ligand interactions and dynamics.^35^, ^36^, ^37^ In future studies, the most accurate docking modelling based on the measured NMR data will be incorporated into our study.

## 5 CONCLUSION

The main purpose of this paper is to find the target of LB. Molecular simulation results show that GLP‐1R is the potential target of LB. SPR detection confirms the interaction between LB and GLP‐1R. Fluorescence spectra and circular dichroism prove that LB has an effect on the conformation of GLP‐1R. In cell experiments, by interfering GPL‐1R, GLP‐1R was proved as the target of LB. Our findings may play a role in the study of anti‐diabetic drugs.

## CONFLICT OF INTEREST

The authors declare that there is no conflict of interest.

## AUTHORS' CONTRIBUTIONS

BN and QC: Design of the study. DY and XS: Completion of the experiments together. All authors took part in interpreting the data and writing the manuscript.

## Supporting information

Fig S1

## References

[1] Grundy SM . Pre‐diabetes, metabolic syndrome, and cardiovascular risk. J Am College Cardiol. 2012;59(7):635‐643. 10.1016/j.jacc.2011.08.080 22322078

[2] Zhuo FU , Gilbert ER , Dongmin L . Regulation of insulin synthesis and secretion and pancreatic Beta‐cell dysfunction in diabetes. Curr Diab Rev. 2013;9(1):25‐53. PMC3934755 22974359

[3] Amer Diabet Assoc . Standards of medical care in diabetes‐2013. Diabetes Care. 2013;36:S11‐S66. 23264422 10.2337/dc13-S011 PMC3537269

[4] Nolan CJ , Damm P , Prentki M . Type 2 diabetes across generations: from pathophysiology to prevention and management. Lancet. 2011;378(9786):169‐181. 21705072 10.1016/S0140-6736(11)60614-4

[5] Diamanti‐Kandarakis E , Duntas L , Kanakis GA , et al. Drug‐induced endocrinopathies and diabetes: a combo‐endocrinology overview. Euro J Endocrinol. 2019;181(2):R73‐R105. 10.1530/EJE-19-0154 31242462

[6] Eurich DT , McAlister FA , Blackburn DF , et al. Benefits and harms of antidiabetic agents in patients with diabetes and heart failure: systematic review. Bmj‐British Med J. 2007;335(7618):497‐501. 10.1136/bmj.39314.620174.80 PMC1971204 17761999

[7] Jung‐Im S . Second‐line glucose‐lowering therapy in type 2 diabetes mellitus. Curr DiabRep. 2019;19(8):54. 10.1007/s11892-019-1171-0 31286271

[8] Aroda VR . A review of GLP‐1 receptor agonists: Evolution and advancement, through the lens of randomised controlled trials. Diab Obesity Metabolism. 2018;20:22‐33. 10.1111/dom.13162 29364586

[9] Drucker DJ . The Cardiovascular biology of glucagon‐like peptide‐1. Cell Metab. 2016;24(1):15‐30. 27345422 10.1016/j.cmet.2016.06.009

[10] Wessel J , Chu AY , Willems SM , et al. Low‐frequency and rare exome chip variants associate with fasting glucose and type 2 diabetes susceptibility. Nat Comm. 2015;6:16. 10.1038/ncomms6897 PMC4311266 25631608

[11] Lee YS , Jun HS . Anti‐diabetic actions of glucagon‐like peptide‐1 on pancreatic beta‐cells. Metabolism‐Clinical Exp. 2014;63(1):9‐19. 10.1016/j.metabol.2013.09.010 24140094

[12] Drucker DJ . Deciphering metabolic messages from the gut drives therapeutic innovation: the 2014 banting lecture. Diabetes. 2015;64(2):317‐326. 25614665 10.2337/db14-1514

[13] Sunil B , Sunder M , Alan C . Evolution of exenatide as a diabetes therapeutic. Curr Diab Rev. 2013;9(2):161‐193. 10.2174/1573399811309020007 PMC3664512 23256660

[14] Werner U . Effects of the GLP‐1 receptor agonist lixisenatide on postprandial glucose and gastric emptying ‐ preclinical evidence. J Diabetes Its Comp. 2014;28(1):110‐114. 10.1016/j.jdiacomp.2013.06.003 23992745

[15] Jones B , Bloom SR , Buenaventura T , Tomas A , Rutter GA . Control of insulin secretion by GLP‐1. Peptides. 2018;100:75‐84. 29412835 10.1016/j.peptides.2017.12.013

[16] Busch RS , Kane MP . Combination SGLT2 inhibitor and GLP‐1 receptor agonist therapy: a complementary approach to the treatment of type 2 diabetes. Postgrad Med. 2017;129(7):686‐697. 28657399 10.1080/00325481.2017.1342509

[17] Mengjun Z , Yunzhen X , Zhou Y , et al. Exendin‐4 enhances proliferation of senescent osteoblasts through activation of the IGF‐1/IGF‐1R signaling pathway. Biochem Biophys Res Comm. 2019;516(1):300‐306. 31256933 10.1016/j.bbrc.2019.06.112

[18] Nauck M . Incretin therapies: highlighting common features and differences in the modes of action of glucagon‐like peptide‐1 receptor agonists and dipeptidyl peptidase‐4 inhibitors. Diabet Obesity Metabolism. 2016;18(3):203‐216. 10.1111/dom.12591 PMC4785614 26489970

[19] Yi FJ , Yi T , Sze‐To C‐M , et al. A Systematic review of the botanical, phytochemical and pharmacological profile of dracaena cochinchinensis, a Plant Source of the Ethnomedicine “Dragon’s Blood”. Molecules 2014;19(7):10650‐10669. 25054444 10.3390/molecules190710650 PMC6270834

[20] Ming HC , Joe‐Sharg L , Khoot‐Peng C , et al. Effect of Sanguis draconis (a dragon\"s blood resin) on streptozotocin‐ and cytokine‐induced β‐cell damage, in vitro and in vivo. Diabetes Res Clin Pract 2011;94(3):417‐425. 21899910 10.1016/j.diabres.2011.08.014

[21] Ruxue Z , Jinrui W , Chunfu WU , Zhengping J , Lingyuan K . Effects of resina draconis on the levels of plasma glucose, insulin and lipids in rats. Trad Chin Drug Res Clin Pharmacol. 2002;13(1):23‐25.

[22] Xianyong YU , Ronghua L , Rongqiong YI , et al. Study of the interaction between N‐confused porphyrin and bovine serum albumin by fluorescence spectroscopy. Spectrochim Acta Part A Mol Spectroscopy. 2011;78(4):1329‐1335. 10.1016/j.saa.2011.01.024 21306939

[23] Ling CF , Jun‐Li W , Yan‐Rui C , Jian‐Ping L . Fluorescent investigation of the interactions between N‐(p‐chlorophenyl)‐N′‐(1‐naphthyl) thiourea and serum albumin: Synchronous fluorescence determination of serum albumin. Anal Chim Acta. 2006;571(2):175‐183. 17723436 10.1016/j.aca.2006.05.002

[24] Wei XL , Bo XJ , Yuanfeng W , Yalong B . Which model based on fluorescence quenching is suitable to study the interaction between trans‐resveratrol and BSA? Spectrochimica Acta Part A Molecular & Biomolecular. Spectroscopy. 2010;75(1):299‐304. 10.1016/j.saa.2009.10.027 19926336

[25] Liu MM , Yang XH , Bai T , et al. PACAP stimulates insulin secretion by PAC(1) receptor and ion channels in beta‐cells. Cell Signal. 2019;61:48‐56. 31085235 10.1016/j.cellsig.2019.05.006

[26] Yang K , Wang M , Zhao YZ , et al. A redox mechanism underlying nucleolar stress sensing by nucleophosmin. Nat Commun. 2016;7:16. 10.1038/ncomms13599 PMC5133708 27886181

[27] Yin Shijin H , Qinglan LJ , Li Yuxin L , et al. Loureirin B, an essential component of Sanguis Draxonis, inhibits Kv1.3 channel and suppresses cytokine release from Jurkat T cells. Cell Biosci. 2014;4(78):1‐8. 25937895 10.1186/2045-3701-4-78 PMC4417528

[28] Annalisa Bordogna A , Pandini A , Bonati L . Predicting the accuracy of protein‐ligand docking on homology models. J Comput Chem. 2011;32(1): 81‐98. 20607693 10.1002/jcc.21601 PMC3057020

[29] Huang RB , Cheng D , Liao SM , et al. The intrinsic relationship between structure and function of the sialyltransferase ST8Sia family members. Curr. Top. Med. Chem. 2017;17(21):2359‐2369. 28413949 10.2174/1568026617666170414150730

[30] Zhou GP , Huang RB . The pH‐triggered conversion of the PrP(c) to PrP(sc.). Curr. Top. Med. Chem. 2013;13(10):1152‐1163. 23647538 10.2174/15680266113139990003

[31] Brunger AT . Version 1.2 of the crystallography and NMR system. Nat. Protoc. 2007;2(11):2728‐2733. 18007608 10.1038/nprot.2007.406

[32] Lane TD , et al. Maltoporin LamB unfolds b hairpins along mechanical stress‐dependent unfolding pathways. Structure. 2017;25:1139‐1144. 28625789 10.1016/j.str.2017.05.010

[33] Xiao T , et al. HIV‐1 fusion inhibitors targeting the membrane‐proximal external region of Env spikes. Nat Chem Biol. 2020;16:529‐537. 32152540 10.1038/s41589-020-0496-y PMC7723321

[34] Chen W , et al. Unidirectional presentation of membrane proteins in nanoparticle‐supported liposomes, Angew. Chem. Int. Ed. Engl. 2019;58(29):9866‐9870. 10.1002/anie.201903093 PMC6660371 30990942

[35] Chou JJ , Mendelsohn ME , Rigby AC , et al. The three‐dimensional structure of the cGMP‐ dependent protein kinase I ‐ α leucine zipper domain and its inter‐ action with the myosin binding subunit. Blood. 2004;104:3539.

[36] Liao SM , et al. Molecular interactions of the polysialytransferase domain (PSTD) in ST8Sia IV with CMP‐sialic acid and polysialic acid required for polysialylation of the neural cell adhesion molecule proteins: An NMR Study. Int J Mol Sci. 2020;21:1590. 10.3390/ijms21051590 PMC7084582 32111064

[37] Huang RB , et al. The cooperative effect between polybasic region (PBR) and polysialyltransferase domain (PSTD) within tumor‐target polysialyltranseferase ST8Sia II. CTMC. 2019;19:2831‐2841. 10.2174/1568026619666191121145924 31755393

