# Supplementary material for: Loureirin B activates GLP‐1R and promotes insulin secretion in Ins‐1 cells
Source: J Cell Mol Med. 2020 Dec 10;25(2):855–66. doi: 10.1111/jcmm.16138 (PMC7812269; doi:10.1111/jcmm.16138)
Supplement: Supplementary file 1 — Fig S1 [file JCMM-25-855-s001.docx]

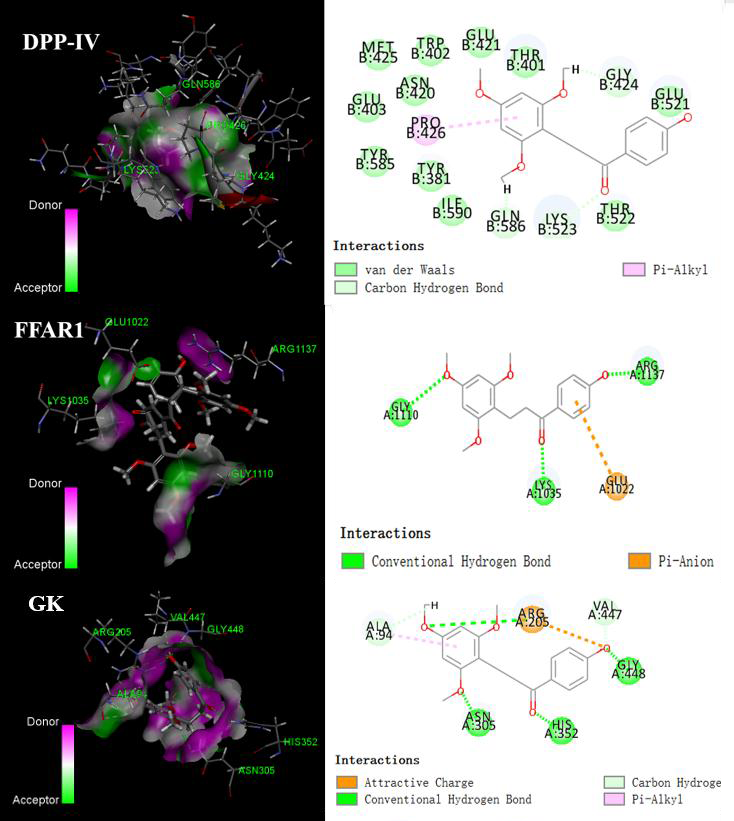


**Figure S1** **Molecular docking diagram of LB interaction with DPP-IV, FFAR1, GK.**

The extracellular domain of GLP-1R was got from the PDB database (4ZGM, http://www.rcsb.org/pdb). The structure of LB was obtained from the PubChem database (https://pubchem.ncbi.nlm.nih.gov). The simulated interaction between LB and GLP-1R was measured by Discovery Studio 4.1 (DS)
